# Supplementary material for: Unveiling Gene Expression Dynamics during Early Embryogenesis in Cynoglossus semilaevis: A Transcriptomic Perspective
Source: Life (Basel). 2024 Apr 15;14(4):505. doi: 10.3390/life14040505 (PMC11050975; doi:10.3390/life14040505)
Supplement: Supplementary file 1 [file life-14-00505-s001.zip › Supplementary Figures.docx]

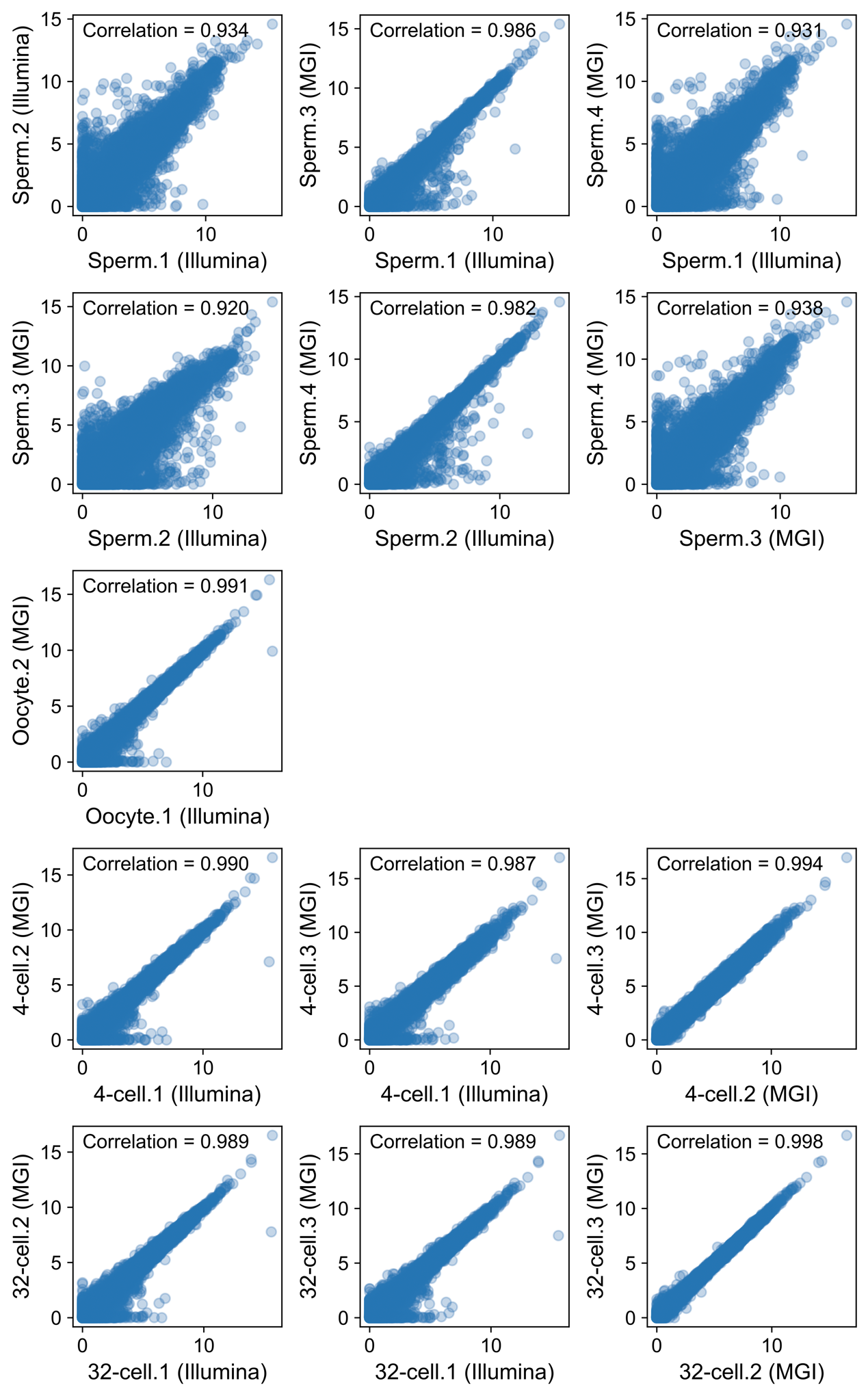


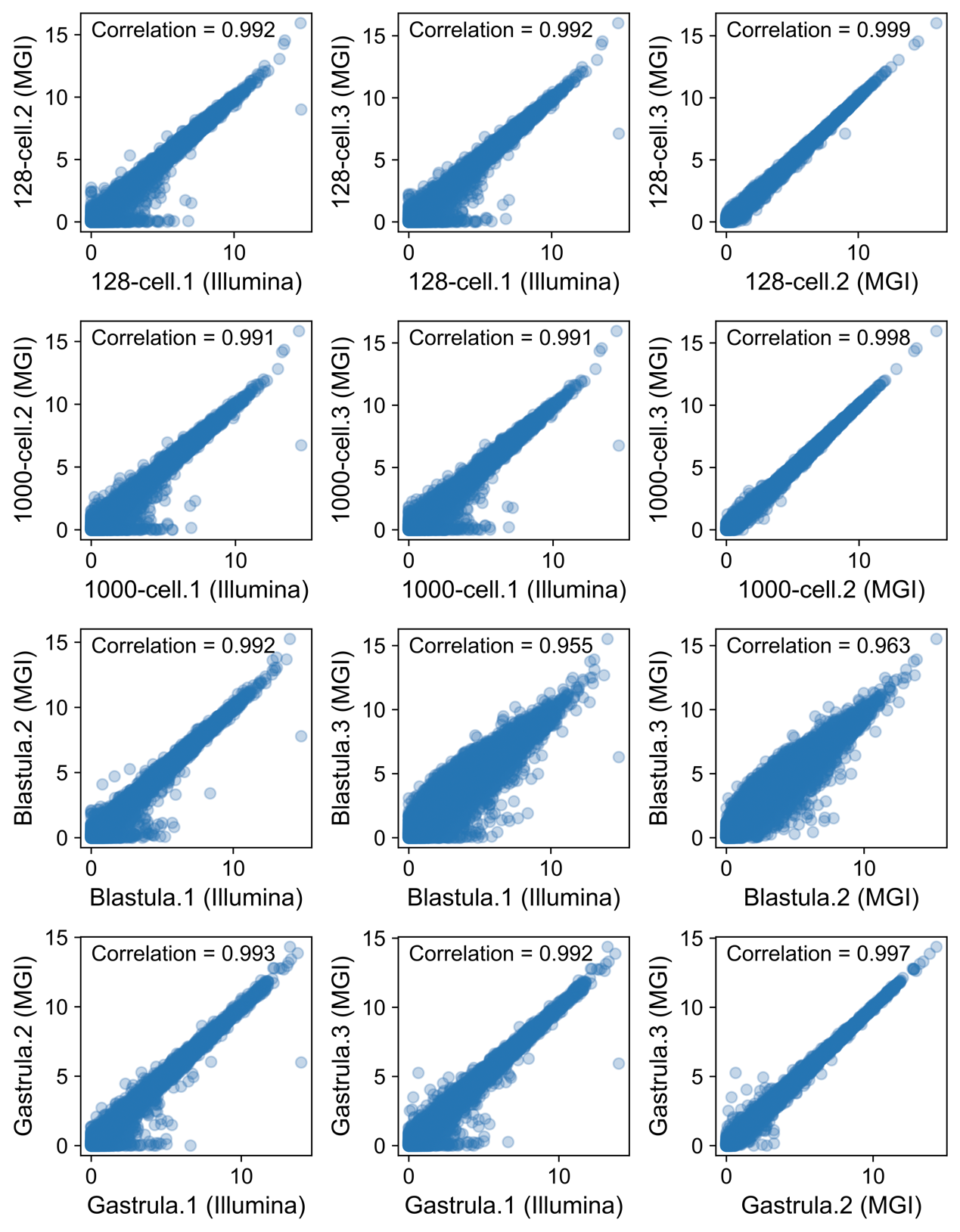


**Figure S1.** Pairwise comparison and Pearson’s correlation of gene expression between biological replicates within the same sample category. The sequencing platform of each replicate is shown in parentheses.


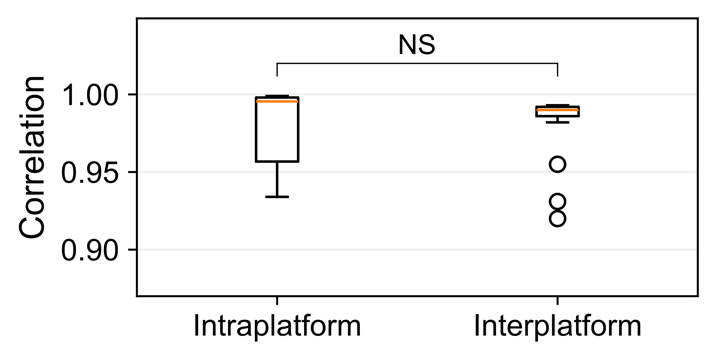


**Figure S2.** Pearson’s correlation coefficient obtained from intra-platform comparisons versus those from inter-platform comparisons. NS: not significant assessed by Student's t-test. The correlation coefficient data for boxplot are from Figure S1.


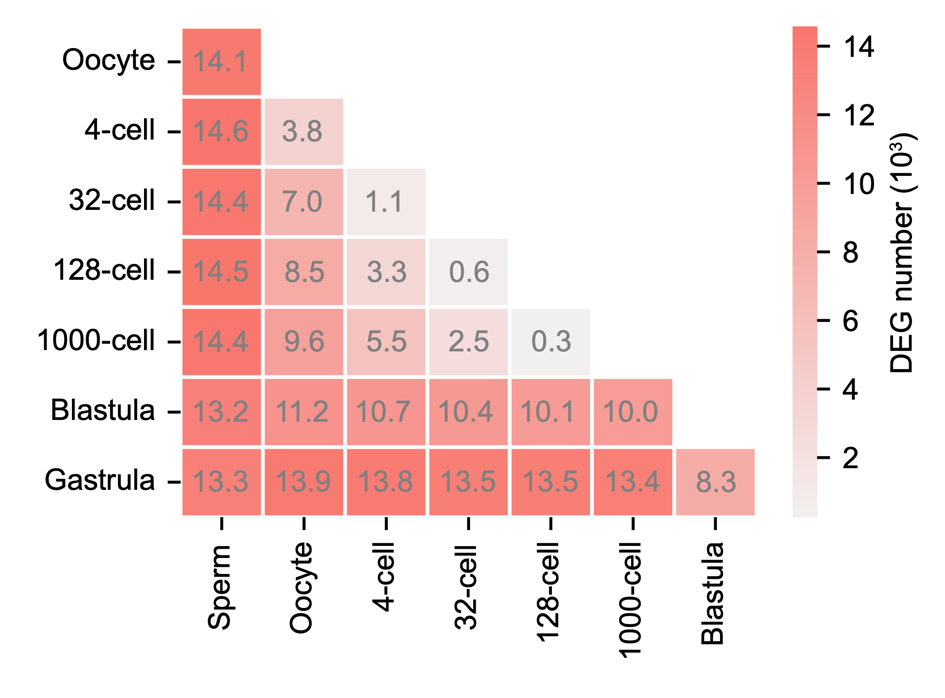


**Figure S3.** Number of differentially expressed genes (DEGs) in pairwise comparison.
